# Inflammatory-Driven Angiogenesis in Bone Augmentation with Bovine Hydroxyapatite, B-Tricalcium Phosphate, and Bioglasses: A Comparative Study

**DOI:** 10.1155/2018/9349207

**Published:** 2018-09-12

**Authors:** Vlad M. Anghelescu, Ioana Neculae, Octavian Dincă, Cristian Vlădan, Claudiu Socoliuc, Mirela Cioplea, Luciana Nichita, Cristiana Popp, Sabina Zurac, Alexandru Bucur

**Affiliations:** ^1^Carol Davila University of Medicine and Pharmacy, Faculty of Dental Medicine, Bucharest, Romania; ^2^Department of Oral and Maxillofacial Surgery, Dan Theodorescu University Hospital of Oral and Maxillofacial Surgery, Bucharest, Romania; ^3^Department of Pathology, Colentina University Hospital, Bucharest, Romania

## Abstract

**Introduction:**

The clinical use of bioactive materials for bone augmentation has remained a challenge because of predictability and effectiveness concerns, as well as increased costs. The purpose of this study was to analyse the ability to integrate bone substitutes by evaluating the immunohistochemical expression of the platelet endothelial cell adhesion molecules, vascular endothelial growth factor, collagen IV, laminin, and osteonectin, in the vicinity of bone grafts, enabling tissue revascularization and appearance of bone lamellae. There is a lack of *in vivo* studies of inflammatory-driven angiogenesis in bone engineering using various grafts.

**Methods:**

The study was performed in animal experimental model on the standardized monocortical defects in the tibia of 20 New Zealand rabbits. The defects were augmented with three types of bone substituents. The used bone substituents were beta-tricalcium phosphate, bovine hydroxyapatite, and bioactive glasses. After a period of 6 months, bone fragments were harvested for histopathologic examination. Endothelial cell analysis was done by analysing vascularization with PECAM/CD31 and VEGF and fibrosis with collagen IV, laminin, and osteonectin stains. Statistical analysis was realized by descriptive analysis which was completed with the kurtosis and skewness as well as the Kruskal-Wallis and Mann-Whitney statistical tests.

**Results:**

The discoveries show that the amount of bone that is formed around beta-tricalcium phosphate and bovine hydroxyapatite is clearly superior to the bioactive glasses. Both the lumen diameter and the number of vessels were slightly increased in favor of beta-tricalcium phosphate.

**Conclusion:**

We can conclude that bone substitutes as bovine bone and beta-tricalcium phosphate have significant increased angiogenesis (and subsequent improved osteogenesis) compared to the bioactive glass. In our study, significant angiogenesis is linked with a greater tissue formation, indicating that in bone engineering with the allografts we used, inflammation has more benefic effects, the catabolic action being exceeded by the tissue formation.

## 1. Introduction

The bone augmentation, used to enhance the bone volume, needed to insert dental implants, can be done with both autologous bone and bone replacement materials. Regardless of the nature of the material, it is necessary to have angiogenesis in order to integrate it into the receptor bed and to maintain its volume. This ensures the presence and proliferation of preosteoblasts [1]. When preosteoblasts differentiate into osteoblasts, they contribute to a much more intense lamellar bone formation around the bone substitute particles.

Inflammation and proinflammatory cytokines (IL-1, IL-4, TNF-*α*, IL-6, IL-5, and prostaglandins) [2] released in the area of bone augmentation are responsible for two opposite effects: bone catabolism and bone regeneration and augmentation [3]. One of the most important regenerating effects of inflammatory cytokines is promoting angiogenesis by stimulating the release of vascular endothelial growth factor (VEGF), newly formed vessels being responsible for revascularization and callus formation [4]. Understanding the inflammatory response helps rational therapeutic intervention in order to maximize benefic effects of inflammation in the healing of engineered tissue [2].

Revascularization is vital for the healing of bone defects, which in turn facilitates the development of osteoid deposition and matrix development in normal bone healing. The link between angiogenic and osteogenic cells is important because in practice, there is a greater need for vascularization where bone grafting materials are used as they are larger, more complex, and require increased control in tissue formation and development [5, 6].

There is still a lack of *in vivo* studies of inflammatory-driven angiogenesis in bone engineering using various grafts, although angiogenesis is a key factor of bone regeneration with crucial role in the success rate of bone augmentation [7].

The purpose of this study was to analyse in animal model the ability to integrate bone substitutes by evaluating the angiogenesis as revealed by immunohistochemical expression of the platelet endothelial cell adhesion molecules (PECAM-1/CD31) and vascular endothelial growth factor (VEGF) and the scaring and bone remodelling process as revealed by immunohistochemical expression of collagen IV, osteonectin, and laminin, for three materials generally used for bone augmentation: bovine hydroxyapatite, betatricalcic phosphate, and bioactive glasses. The evaluated proteins are markers of the endothelium of newly formed blood vessels in the vicinity of the bone grafts.

## 2. Materials and Methods

### 2.1. Animal Model

The study was conducted at the Cantacuzino National Research and Development Institute for Microbiology and Immunology Bucharest, on 20 mature rabbits (6–9 months old) from the New Zealand White breed (10 males and 10 females) with an average weight of about 2.5 kg. The study protocol and experimental scheme were approved by the Ethics Commission for working with laboratory animals of INCDMI Cantacuzino Bucharest in conformity with ISO 10993-2 having the number of protocol CE/37/23.02.2015. Experimental animals have been housed in special enclosures with a constant temperature between 18°C and 24°C and 55% humidity and fed a diet according to the standards. The study specimens were randomly divided according to the material with which the bone augmentation was performed in three groups (group A, group B, and group C). Surgical interventions were performed with general anaesthesia with a 3 ml solution containing a mixture of 2.3 ml of ketamine and 0.7 ml of xilazine. It was divided into 2 equal doses of 1.5 ml intramuscular injections, administered at 3-minute intervals. The control group consisted of two animals whose defects were augmented with autograft obtained by grinding the bone from the intracortical defect bone drilling.

**Figure 1:**
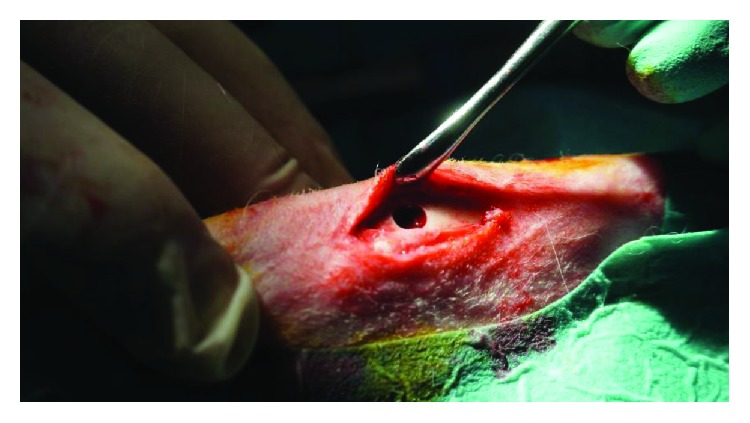
Bone circular defect in the proximal tibia after drilling.

The surgical procedure consisted in drilling of several cortical cavities in the proximal region of the tibia using rotary instruments under cooling. For the exposure of the tibial cortex, incisions of approximately 5/2 centimetres were performed and dissection of the muscular inserts from the bony plane was performed. The medial face of the tibia, in the proximal third, was exposed to a length of 6 cm. After identifying and sectioning the periosteum, it was partially cut off, respecting its integrity, for the subsequent coverage of the augmented bone defect. Using a tubular drill of 4 mm internal diameter and 2 mm outer diameter, an intracortical geode in the central area of the bone was performed to each tibia, obtaining a bone defect having the same diameter as the tubular drill (Figure 1). The bone augmentation materials used in the present study have been commercially obtained. The used bone substituents were beta-tricalcium phosphate (Cerasorb®, Curasan regenerative materials) which is a fully synthetic bioactive bone substitute, bovine hydroxyapatite (BioOSS, Geistlich Biomaterials) which consists of pure mineral content of bovine bone, and bioactive glasses (Perioglass, Novabone) having the composition 45% SiO_2_ (silica dioxide), 24.5% CaO (calcium oxide), 24.5% NaO_2_ (sodium oxide), and 6% P_2_O_5_ (phosphorus pentoxide). Six defects were augmented with bovine hydroxyapatite; another 6 defects were augmented with *β*-tricalcium phosphate (TCP) and another 6 with bioactive glasses. In the two remaining defects, the augmentation was done with the bone obtained by drilling the intracortical geodes, which was subjected to a grinding process. Against augmenting biomaterials, nonresorbable PTFE-based membranes were applied to obtain a barrier to connective tissue. After application of the membranes, a wound suture was made in three planes (periost, muscle tissue, and tegument). Ketoprofen 3 mg/kg was postoperatively administered to animals for three days.

Animals were euthanized 180 days after bone augmentation intervention, by intravenous administration of phenobarbital 200 mg/ml. The tissue fragments were sampled from the augmented areas, which were subsequently histopathologically examined.

Tricalcium phosphate is a bone substitute widely used due to its good biocompatibility, biological safety, virtually unlimited availability, and ease of sterilization [8]. Hydroxyapatite, although has a good biocompatibility, has a limited ability to degrade and absorb within the organism [9]. Bioactive glasses are an alternative to inert implant materials, bonding with host bone to create a stable implant [10].

### 2.2. Immunohistological Labeling

The specimens were decalcified in a HCL/EDTA solution for 72 hours after being immersed in 10% buffered formalin for 24 hours. Tissue fragments were selected so that the soft tissue, the graft, and its interface with the receptor bed could be studied. After decalcification, the fragments were routinely processed on a Leica ASP200S tissue processor for ethanol 70° for 90 min at 40°C, ethanol 80° for 105 min at 40°C, ethanol 96° for 105 min at 40°C, ethanol 100° for 60 min at 40°C, ethanol 100° for 90 min at 40°C, ethanol 100°C for 90 min at 40°C, xylene for 2 hours at 52°C, paraffin 58°C 1 hour, paraffin 58°C 2 hours, and paraffin 58°C 3 hours. The tissue fragments were embedded in paraffin blocks using a Thermo Fisher Microm EC 1150 H embedding station. Sections of 3-micron thickness were cut using a Leica RM 2265 rotary microtome. Slides were routinely stained with hematoxylin and eosin (HE). Immunohistochemical (IHC) tests were performed for PECAM-1/CD31 (Thermo Fisher Scientific monoclonal antibody, JC/70A clone, high-temperature antigen recovery using 0.01 M citrate recovery solution pH 6 for 15 minutes, 1 : 50 dilution for 60 minutes, and the mouse-specific HRC/DAB (ABC) Detection IHC Kit (Abcam, AB64259)), VEGF (Thermo Fisher Scientific, monoclonal antibody, MA5-12184 clone, high-temperature antigen recovery using 0.01 M citrate recovery solution pH 6 for 15 minutes, 1 : 40 dilution for 60 minutes, and the mouse-specific HRC/DAB (ABC) Detection IHC Kit (Abcam, AB64259)), laminin (Thermo Fisher Scientific, polyclonal antibody, PA1-16730 clone, high-temperature antigen recovery using 0.01 M citrate recovery solution pH 6 for 15 minutes, 1 : 50 dilution for 60 minutes, and the mouse-specific HRC/DAB (ABC) Detection IHC Kit (Abcam, AB64259)), collagen IV (Thermo Fisher Scientific, polyclonal antibody, PA1-28534 clone, high-temperature antigen recovery using 0.01 M citrate recovery solution pH 6 for 15 minutes, 1 : 400 dilution for 60 minutes, and the mouse-specific HRC/DAB (ABC) Detection IHC Kit (Abcam, AB64259)), osteonectin (Thermo Fisher Scientific, polyclonal antibody, PA1-16730 clone, high-temperature antigen recovery using 0.01 M citrate recovery solution pH 6 for 15 minutes, 1 : 50 dilution for 60 minutes, and the mouse-specific HRC/DAB (ABC) Detection IHC Kit (Abcam, AB64259)), and collagen IV (Thermo Fisher Scientific, monoclonal antibody, MA5-17180 clone, high-temperature antigen recovery using 0.01 M citrate recovery solution pH 6 for 15 minutes, 1 : 500 dilution for 60 minutes, and the mouse-specific HRC/DAB (ABC) Detection IHC Kit (Abcam, AB64259)).

Microscopic examination of the slides was performed using a Nikon Eclipse 80i microscope, and the photographs were obtained using a digital camera attached to a computer.

Endothelial cell analysis was done by analysing endothelial cell adhesion to platelets with the marker PECAM/CD31 and the proangiogenetic effect with VEGF that is strongly expressed by endothelial cells.

The amount and distribution of fibrosis were evaluated on collagen IV, osteonectin, and laminin stains.

Immunohistochemical controls were used to assess the efficacy and specificity of reactions by negative reagent controls and internal negative tissue controls.

The number of vessels and their lumen around the de novo formed bone particles was evaluated. In sections marked with anti-CD31 and VEGF antibodies, the number of vessels and the lumen diameter were comparatively analysed by visual analysis. Data were described by comparison between the three materials used, bovine bone mineral, beta-tricalcium phosphate, and bioactive glasses (Perioglass).

Histologic measuring of the new bone formation was semiquantitatively evaluated and correlated with the vessel formation around the augmented materials

### 2.3. Statistical Analysis

The statistical software SPSS 22 was used for data analysis. The descriptive analysis was used for primary assessment. Kurtosis and skewness tests were used to assess the asymmetrical distribution of the data. Also, the nonparametric tests for independent batches Kruskal-Wallis and Mann-Whitney were used.

For the histologic evaluation of bone formation, the nonparametric test for the independent batches Kruskal-Wallis was used. Kolmogorov-Smirnov and Shapiro-Wilk tests were performed to assess the normality.

## 3. Results

**Figure 2:**
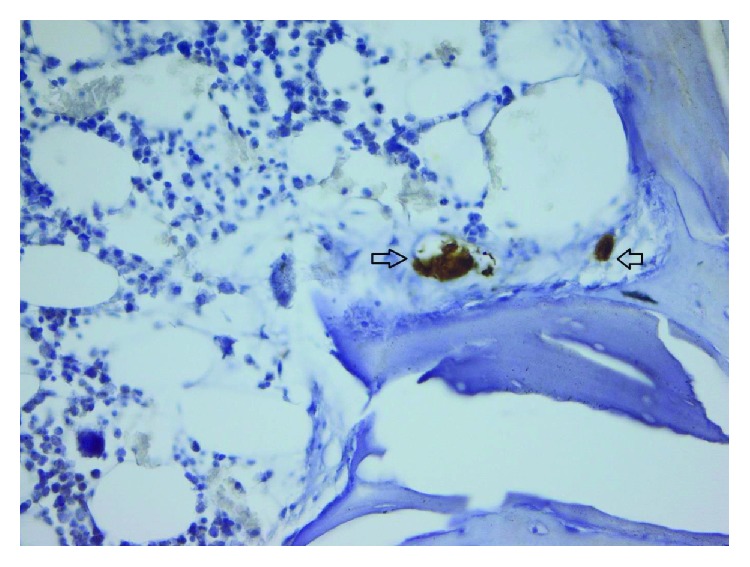
40x bovine hydroxyapatite PECAM.

**Figure 3:**
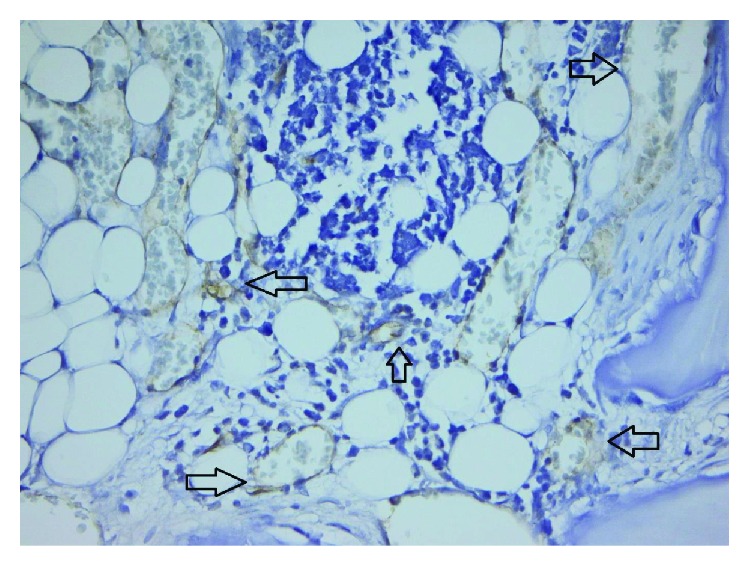
40x bovine hydroxyapatite PECAM −1/CD31 reactive endothelium.

**Figure 4:**
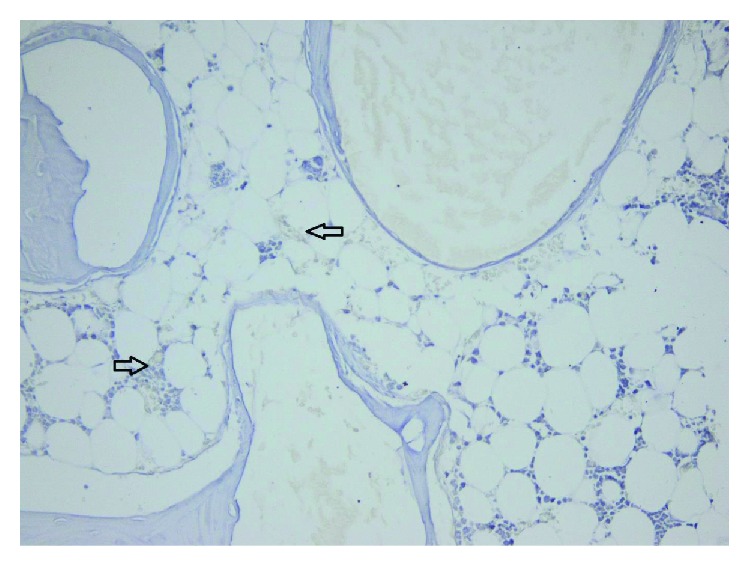
40x TCP PECAM-1/CD31 reactive endothelium.

**Figure 5:**
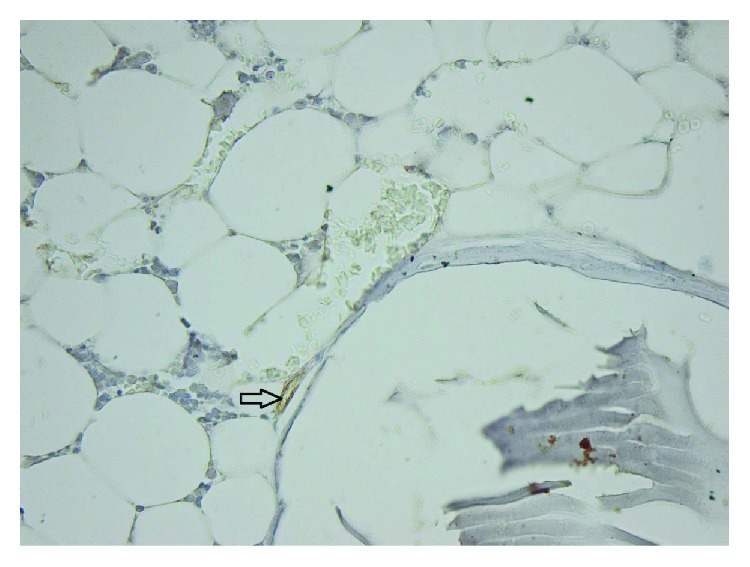
40x TCP PECAM-1/CD31 reactive endothelium.

**Figure 6:**
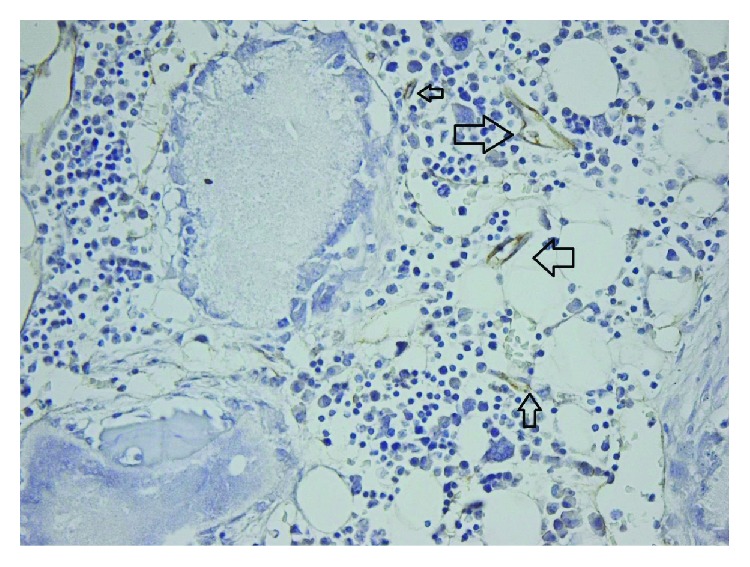
40x bioglass PECAM-1/CD31 reactive endothelium.

**Figure 7:**
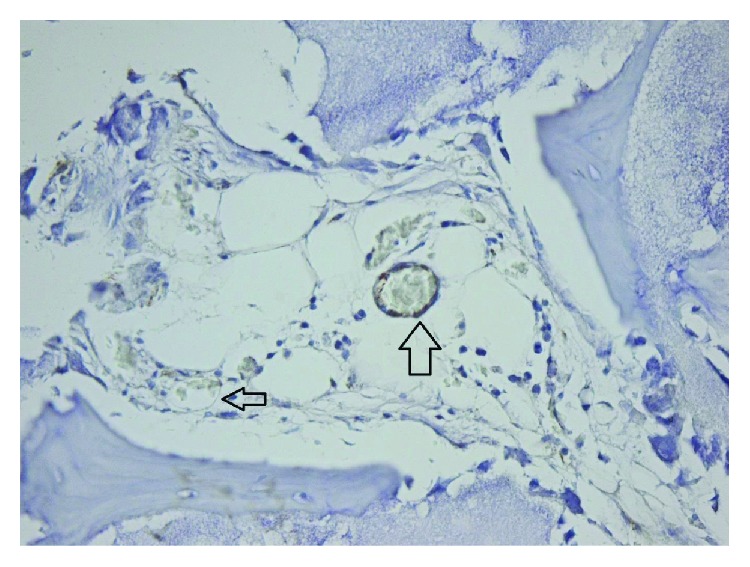
Bioglass PECAM-1/CD31 reactive endothelium.

**Figure 8:**
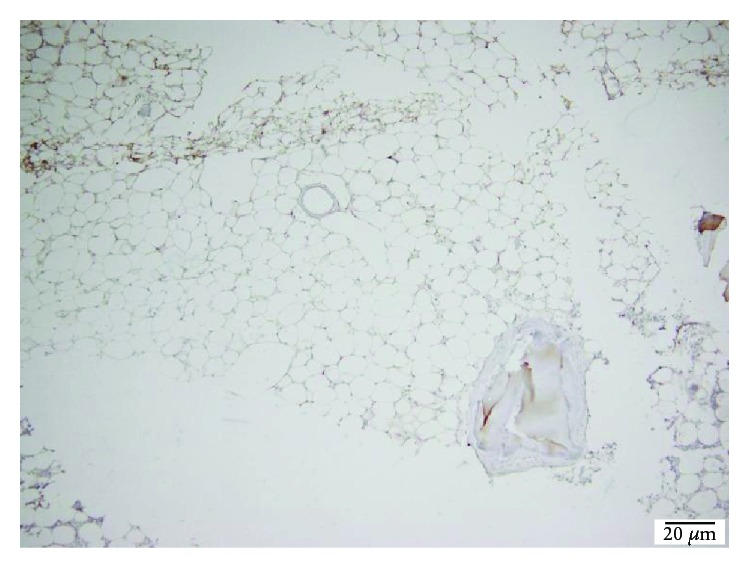
Bioglass 100 × VEGF reactive endothelium.

**Figure 9:**
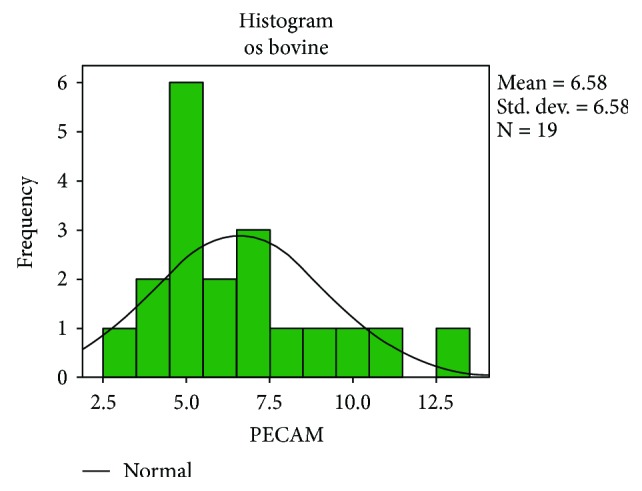
Histogram standard deviation bovine bone.

**Figure 10:**
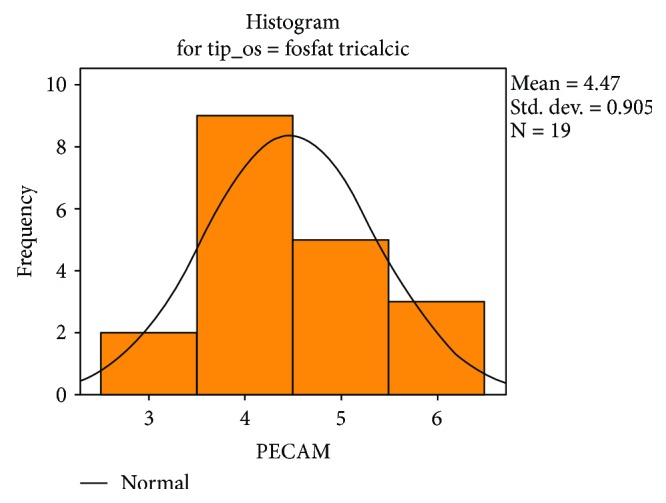
Histogram standard deviation B-tricalcium phosphate.

**Figure 11:**
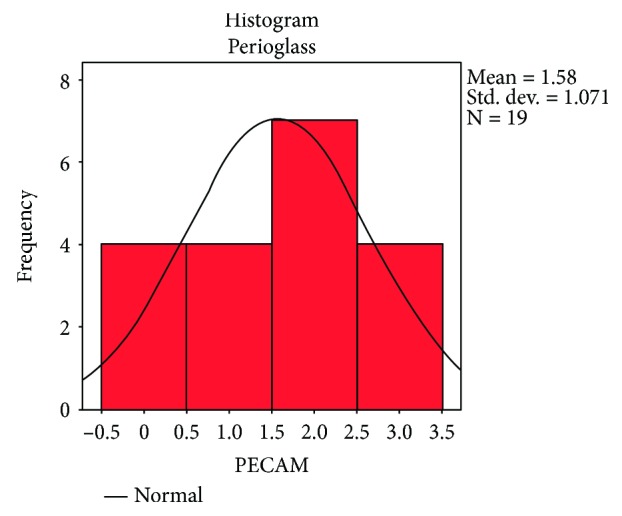
Histogram standard deviation bioactive glass.

Following the quantitative descriptive analysis of the distribution of CD31- and VEGF-positive vessels around the augmentation material, it was noted that we maintain an increased average of the number of vessels in the bovine batch group 6.58 ± 0.59 (Figures 2 and 3) and the tricalcium phosphate 4.47 ± 0.2 (Figures 4 and 5) compared to the lot where the bioactive glass 1.58 ± 0.246 was used (Figures 6–8) (Table 1). The kurtosis and skewness tests reveal an asymmetric distribution to the right in the case of the first batch (Figure 9) and distribution to the left for the other two batches (Figures 10 and 11) with a platrictic aspect of the curve.

**Figure 12:**
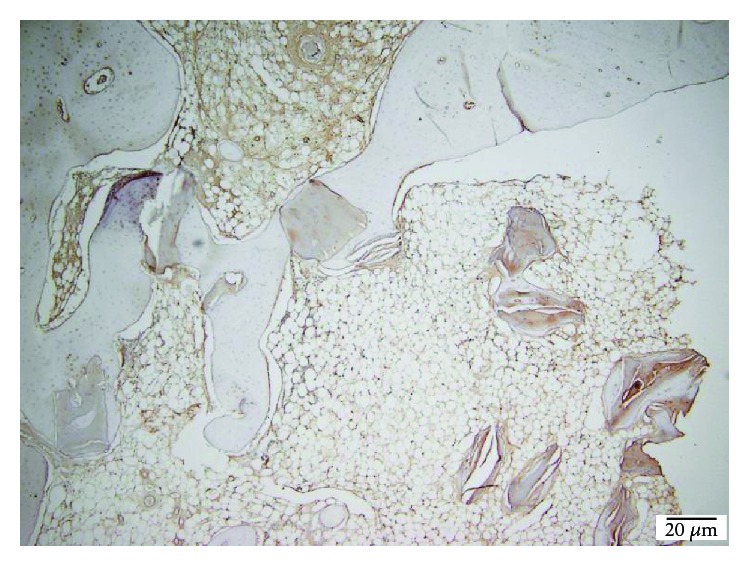
TCP 100x collagen IV stain showing moderate fibrosis.

**Figure 13:**
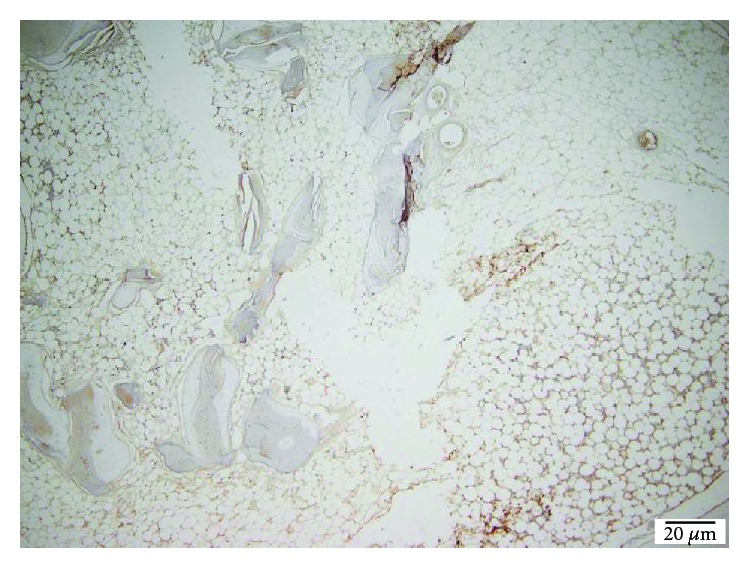
TCP 40x laminin stain showing moderate scaring.

The amount of fibrosis, evaluated on collagen IV and laminin stains, showed no statistically significant differences between the three groups and, also, no differences comparing with the control group (Figures 12 and 13).

**Figure 14:**
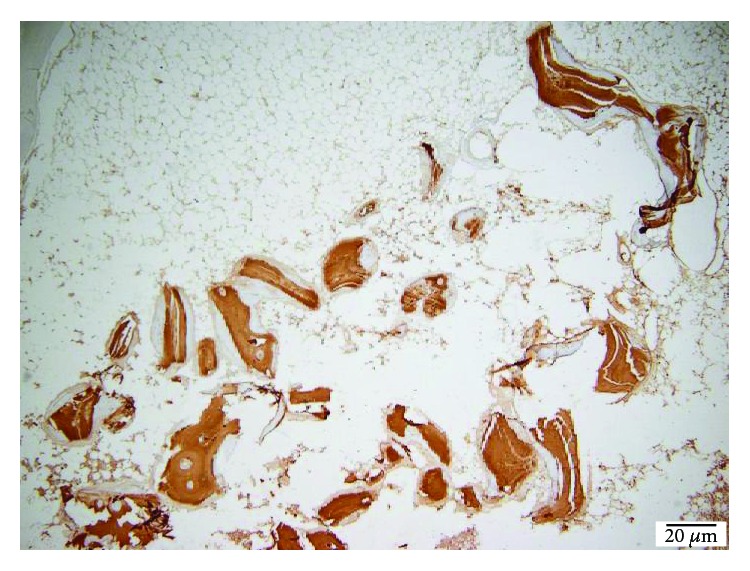
TCP 40x osteonectin stain showing intense bone remineralization.

Bone remodelling and remineralization, as evaluated on osteonectin stain, were also similar in all groups (control group, beta-tricalcium phosphate, bovine hydroxyapatite, and bioactive glasses groups) (Figure 14).

**Figure 15:**
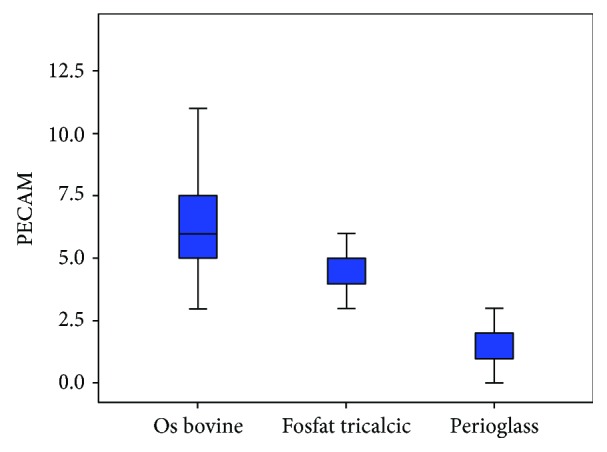
Representation of the mean number of blood vessels.

Using the Kruskal-Wallis test, the average score in the first batch was 43.7 followed by 32.9 for tricyclic phosphate group and only 10.32 for the bioactive glass lot. From the statistical analysis, we obtained the significant difference between the three groups, chi square = 40.0 to 2 degrees of freedom with *p* < 0.001 (Figure 15) (Table 2).

Applying the Mann-Whitney test, wave *U* = 79 (*N*1 = 19, *N*2 = 19) was obtained at a significance level *p* = 0.002 < 0.05 (Tables 3 and 4).

From the above analysis, statistically significant differences are observed for all three pairs. This complements the discoveries in which the amount of bone that is formed around beta-tricalcium phosphate and bovine hydroxyapatite is clearly superior to the bioactive glasses. Both the lumen diameter and the number of vessels were slightly increased on the studied slides in favour of beta-tricalcium phosphate.

Using the Kruskal-Wallis test, the average score in the first batch was 100.4, followed by 72.73 for the tricyclic phosphate group and only 34.01 for the bioactive glass batch. From the statistical analysis, we obtained the significant difference between the three groups, chi square = 59.323*n* at 2 degrees freedom with *p* < 0.001 (Tables 5 and 6). Following normality tests, Kolmogorov-Smirnov and Shapiro-Wilk demonstrated that the only batch that respects normality is the autologous control bone (Table 7).

This complements the findings in which the amount of bone that forms around beta-tricalcium phosphate and bovine hydroxyapatite is clearly superior to bioactive glasses.

## 4. Discussions

The purpose of this study was to analyse the ability to integrate bone substitutes by evaluating the IHC expression of the platelet endothelial cell adhesion molecules (PECAM-1/Cd31), VEGF, collagen IV, laminin, and osteonectin for the three materials used for augmentation: bovine hydroxyapatite, beta-tricalcium phosphate, and bioactive glasses. Angiogenesis is the witness of inflammatory processes with both catabolic and anabolic effects, and this *in vivo* study used CD31 as an indicator of intensity of inflammatory-driven angiogenesis.

The cluster of differentiation 31 (CD31), also known as the platelet endothelial cell adhesion molecule (PECAM-1), plays a key role in removing aged neutrophils from the body. It is found on the surface of platelets, monocytes, neutrophils, and some types of T cells. The encoded protein is a member of the immunoglobulin superfamily and is probably involved in leukocyte transmigration, angiogenesis, and integrin activation. CD31 and VEGF are mainly used to demonstrate the presence of endothelial cells in histological tissue sections [11, 12].

In a bone defect, angiogenesis is a mandatory process in order to obtain osteogenesis [13–16]. Osteogenesis occurs most frequently through osteoconductivity mechanism. This is a three-dimensional process in which the biomaterial for augmentation acts as a matrix for the capillary proliferation, and the migration of proosteoblasts forms the adjacent tissue [17, 18]. Osteoconductive materials cannot be used in the reconstruction of large bone defects, since bone formation is limited by the distance on which the replacement material is applied [19, 20]. In order to reconstruct large defects, it is preferable to use osteoinductive materials [21].

The number of vessels and lumens quantified in the studied sections stained with the CD31 and VEGF immunomarkers was higher in bovine bone graft rabbits without a noticeable difference as compared to beta-tricalcium phosphate. Around the Perioglass material, we noticed the smallest number of vessels and the smallest amount of bone, this being consistent with other studies [22, 23]. Therefore, the superiority of the new bone formation in the case of hydroxyapatite could be justified by its osteoconductive property as well as by the larger quantity of vessels in which it was present.

Zengin et al. and Matsumoto et al. reported the existence of a cell population between the tunica media and the adventitia of adult vessels, which can behave as immature endothelial cells or osteoprogenic cells. Increased amount of CD31+ cells could induce an increase in the number of pericytes which probably contributes to osteoblast population growth [14, 24, 25]. Another study reported by Rabie has determined that anterior vascularization of the graft increases the metabolism of osteoblasts. Failure of this process initiates chondroblast and osteoclast activity and subsequent graft tissue absorption [26].

In our study, significant angiogenesis is linked with a greater tissue formation, indicating that in bone engineering with the allografts we used, inflammation has more benefic effects, the catabolic action being exceeded by the tissue formation.

## 5. Conclusions

Based on the results of this study, we can conclude that bone substitutes as bovine bone and beta-tricalcium phosphate have significant increased angiogenesis (and subsequent improved osteogenesis) comparing with Perioglass. There is a tendency towards better activity of bovine bone comparing with beta-tricalcium phosphate, but data are not statistically significant. Bovine bone (hydroxyapatite) has more prominent osteogenic potential because it forms a matrix that allows capillaries to develop, facilitating bone formation due to its osteoconductive property.

## Figures and Tables

**Table 1:** Descriptive analysis blood vessel representation.

	Bone type		Statistic	Std. error
PECAM	Bovine bone	Mean	6.58	0.599
		Median	6.00	
		Std. deviation	2.610	
		Minimum	3	
		Maximum	13	
		Skewness	1.063	0.524
		Kurtosis	0.686	1.014
	*β*-Tricalcium phosphate	Mean	4.47	0.208
		Median	4.00	
		Std. deviation	0.905	
		Minimum	3	
		Maximum	6	
		Skewness	0.339	0.524
		Kurtosis	−0.499	1.014
	Bioactive glass	Mean	1.58	0.246
		Median	2.00	
		Std. deviation	1.071	
		Minimum	0	
		Maximum	3	
		Skewness	−0.229	0.524
		Kurtosis	−1.102	1.014

**Table 2:** Kruskal-Wallis test ranks.

	Bone type	*N*	Mean rank
PECAM	Bovine bone	19	43.74
	*β*-Tricalcium phosphate	19	32.95
	Bioactive glass	19	10.32
	Total	57	

**Table 3:** *β*-Tricalcium phosphate and bioactive glass Mann-Whitney test statistics.

	Bone type	*N*	Mean rank	Sum of ranks
PECAM	*β*-Tricalcium phosphate	19	28.79	547.00
	Bioactive glass	19	10.21	194.00
	Total	38		

**Table 4:** Bovine bone and bioactive glass Mann-Whitney test statistics.

	Bone type	*N*	Mean rank	Sum of ranks
PECAM	Bovine bone	19	28.89	549.00
	Bioactive glass	19	10.11	192.00
	Total	38		

**Table 5:** Kruskal-Wallis test.

Statistic test	Aria
Chi square	59.323
df	2
Asymp. sig.	0.000

**Table 6:** Values of newly formed bone on every type of material.

	Bone type	*N*	Mean rank
Aria	HA	51	100.43
	BTCP	50	72.73
	Perioglass	41	34.01
	Total	142	

**Table 7:** Normality test in which it can be observed that the only substitution material that respects normality is the autologous bone.

Bone type	Kolmogorov-Smirnov^a^			Shapiro-Wilk		
	Statistic	df	Sig.	Statistic	df	Sig.
TCP	0.277	51	0.000	0.634	51	0.000
Bovine	0.171	50	0.001	0.804	50	0.000
Perioglass	0.239	41	0.000	0.770	41	0.000
Autologous	0.129	10	0.200^∗^	0.963	10	0.816

## Data Availability

The data used to support the findings of this study are available from the corresponding author upon request.
